# Lectin-Mediated Binding of Engineered *Lactococcus lactis* to Cancer Cells

**DOI:** 10.3390/microorganisms9020223

**Published:** 2021-01-22

**Authors:** Tina Vida Plavec, Abida Zahirović, Petra Zadravec, Jerica Sabotič, Aleš Berlec

**Affiliations:** 1 Department of Biotechnology, Jožef Stefan Institute, Jamova 39, 1000 Ljubljana, Slovenia; tina.plavec@ijs.si (T.V.P.); abida.zahirovic@ijs.si (A.Z.); petrazadravec@gmail.com (P.Z.); jerica.sabotic@ijs.si (J.S.); 2 Faculty of Pharmacy, University of Ljubljana, Aškerčeva 7, 1000 Ljubljana, Slovenia; 3 Lek d.d., Kolodvorska 27, 1234 Mengeš, Slovenia

**Keywords:** *Lactococcus lactis*, colorectal cancer, targeting, lectins, B subunit of Shiga toxin, *Clitocybe nebularis* lectin

## Abstract

Lectins have been increasingly utilized as carriers for targeted drug delivery based on their specific binding to glycans located on mammalian cells. This study employed two lectins, B subunit of bacterial Shiga holotoxin (Stx1B) and fungal *Clitocybe nebularis* lectin (CNL), for surface display on the lactic acid bacterium *Lactococcus lactis*. The specific adhesion of these engineered, lectin-displaying *L. lactis* to cancer cells was evaluated. The expression and surface display of both lectins on *L. lactis* were demonstrated by western blotting and flow cytometry, respectively. MTS assays revealed that recombinant Stx1B had no effect on Caco-2 cell viability at concentrations of ≤25 µg/mL, whereas CNL was non-toxic even at relatively high concentrations of ≤250 µg/mL. Stx1B bound to Caco-2, HT-29 and HeLa cells after 1 h of incubation. CNL bound to Caco-2 cells and recognized several glycoproteins in HT-29 and Caco-2 cell homogenates of which a 70 kDa protein predominated. Confocal microscopy revealed adhesion of Stx1B-displaying *L. lactis* to HeLa, Caco-2, and, to a lesser extent, HT-29 cells; CNL-displaying *L. lactis* showed a relatively similar level of adherence to HT-29 and Caco-2 cells. Thus, lectin-displaying *L. lactis* might serve as a carrier in targeted drug delivery when coupled to a therapeutic moiety.

## 1. Introduction

Altered glycosylation patterns and overexpression of specific carbohydrate epitopes are hallmarks of many cancers [1]. Changes in the oligosaccharide structures of tumor-associated glycoproteins or glycolipids include increased N-glycan branching, a higher O-glycan density, and the generation of truncated versions or modification of terminal glycan molecules through sialylation and fucosylation [2]. These alterations can be exploited for targeted therapy, which is one of the goals of precision medicine. Carbohydrate receptors or patterns on the cell surface mediate intercellular interactions [3] and can be recognized by lectins, a heterogeneous group of proteins and glycoproteins with a selective affinity for carbohydrates [4]. Lectins are found in a diversity of organisms ranging from viruses and plants to humans. Human endogenous lectins are involved, through their specific interactions with complex carbohydrates, in numerous physiological and pathological processes, such as intracellular trafficking, recognition processes, cell homing, endocytosis, phagocytosis, and inflammation [5].

Exogenous lectins have been exploited for their directed binding to cell surfaces for targeted cancer therapy, i.e., targeted delivery of anticancer drugs [6,7,8]. For this purpose, a lectin-targeting moiety is conjugated to an anti-cancer agent, such as a monoclonal antibody, peptide, or small chemotherapeutic molecule [9,10]. Since carbohydrate structures are altered during the progression of cancer, lectins can distinguish between cell subsets and enable more precise recognition of cancer cells compared to other ligands currently used in active drug delivery systems [11].

We propose the display of lectin as targeting moiety on the host bacteria, which can be further engineered to produce therapeutic molecules, analogous to functionalization of drug-loaded nanoparticles with lectins [12]. Bacteria, such as *Salmonella typhimurium*, have been suggested as an alternative treatment for cancer due to their tropism for anaerobic conditions that are present in central hypoxic regions of tumor tissues [13,14]. A surface-displayed lectin can enhance this intrinsic tumor-homing ability of bacteria via binding to glycoproteins or glycolipids on cancer cells. Such engineered bacteria can be used to facilitate targeted cancer therapy. The feasibility of this strategy is supported by the fact that bacteria use their surface lectins, named adhesins, to attach to cells (e.g., in the gastro-intestinal tract) [15]. In contrast to pathogenic bacteria, the lactic acid bacterium *Lactococcus lactis* (*L. lactis*) is used in food production and is considered a safe organism [16]. Furthermore, it is used as a vector for the mucosal delivery of antigens [17] and therapeutic proteins [18,19,20] as well as for proteins capable of cytokine or chemokine binding [21,22] and toxin removal [23,24].

In this study, two lectins, the B subunit of Shiga holotoxin (Stx1B) and *Clitocybe nebularis* lectin (CNL), have been applied to target *L. lactis* to cancer cells. Stx1B binds to cells by recognizing glycosphingolipid globotriaosylceramide (Gb3, also CD77) on their surface [25]. Gb3 is over-expressed in inflammatory conditions [26] and in various cancer cell lines, including breast, pancreatic, and colon cancer cells [27]. Affinities in the nanomolar range have been observed between Stx1B and cancer cells [28]. CNL from the clouded agaric mushroom (*C. nebularis*) [29] is a β-trefoil-type hololectin that specifically binds N,N’-diacetyllactosediamine (LacdiNAc disaccharide). While LacdiNAc is not generally found in healthy mammalian or human tissues [30], it has been reported in different types of cancers [31]. Increased amounts of LacdiNAc have been demonstrated in the N-glycans of tumor markers, particularly of prostate-specific antigen [32], endonuclease I from a pancreatic cancer cell line [33], and erythropoietin from ovarian cancer cells [34]. Additionally, LacdiNAc has been implicated in the pathogenesis of colorectal cancer in which upregulated transcript levels of β4-N-acetylgalactosaminyltransferase (the enzyme involved in LacdiNAc biosynthesis) have been demonstrated [35]. The overexpression of this enzyme increased cell adhesion to extracellular matrix, migration, and invasion in a human colon cancer cell line HCT116 as well as promoted tumor growth and metastasis in nude mice [35].

Here, we engineered *L. lactis* to display two lectins, Stx1B and CNL, on its surface and concomitantly express fluorescent protein for detection. We verified the presence of lectins’ target sites on HeLa, HT-29 and Caco-2 cells and demonstrated lectin-mediated adhesion of engineered bacteria to these cells.

## 2. Materials and Methods

### 2.1. Cell Lines and Culturing

Caco-2 (ATCC HTB-37) and HeLa (ATCC CCL-2) cells were cultured and passaged in Dulbecco’s modified Eagle’s medium (Gibco, Thermo Fisher Scientific, Waltham, MA, USA). HT-29 (ATCC HTB-38) was cultured in McCoy’s 5A Modified Medium (ATCC). Both types of cell culture media were supplemented with 10% (*v*/*v*) fetal bovine serum (Gibco) and 1% penicillin-streptomycin (Gibco). For Caco-2 cultures, HEPES (25 mM; Gibco) and 1% minimum essential medium non-essential amino acid solution (Gibco) were added. All cell lines were incubated, maintained, and cultured at 37 °C with 5% CO_2_. All cell lines tested negative for mycoplasma.

### 2.2. SDS-PAGE and Western Blotting

Whole-cell lysates were prepared as follows. HT-29 and Caco-2 cells were seeded onto 6-well plates, and upon reaching confluency, the medium was aspirated and the cell monolayer was gently washed with Dulbecco’s PBS (DPBS, Gibco). The cells were detached and collected in DPBS, transferred to tubes, and centrifuged at 13,523× *g* for 20 min 4 °C. Following supernatant aspiration, RIPA lysis buffer (50 mM Tris/HCl pH 8.0, 150 mM NaCl, 1% Triton-100, 0.5% Na-deoxycholate, 0.1% SDS, 1 mM EDTA) with protease inhibitor was added to the pellet and incubated for 30 min on ice. The pellet was then centrifuged at 16,000× *g* for 20 min at 4 °C, and the protein in the supernatant was collected and stored at −80 °C. Protein concentration was determined by DC Protein Assay (Bio-Rad), and 30 μg of proteins were loaded onto the gel. SDS-PAGE was performed with a Mini-Protean II apparatus (Bio-Rad). Samples were mixed with 2× Laemmli sample buffer and dithiothreitol, and denatured by heating to 100 °C before loading [36]. The Page Ruler Plus (Thermo Fisher Scientific) pre-stained standards were used for molecular weight comparisons. The proteins were transferred to nitrocellulose membranes (GE Healthcare Life Sciences, Marlborough, MA, USA) using semi-dry transfer with a protocol for 1.5 mm gels (Trans-Blot Turbo Blotting System; Bio-Rad). The membrane was blocked with 5% skim dried milk in Tris-buffered saline (TBS) with 0.05% Tween-20 (TBST; 50 mM Tris-HCl, 150 mM NaCl, 0.05% Tween 20, pH 7.5) and incubated overnight at 4 °C with recombinant *E. coli*-expressed CNL [29] diluted at 1:1200 (5 μg/mL) in 5% skim dried milk in TBST. Following three washes with TBST, the membrane was incubated for 1 h with rabbit polyclonal anti-CNL (1:2000) in 5% skim dried milk in TBST. The detection was performed using horseradish peroxidase-conjugated goat anti-rabbit IgG (1:5000, Jackson ImmunoResearch) and Lumi-Light chemiluminescent reagent (Roche). Images were acquired using a ChemiDoc MP imaging system (Bio-Rad). Glyceraldehyde 3-phosphate dehydrogenase (GAPDH) was used as a loading control and was detected by rabbit monoclonal anti-GAPDH (1:5000; Proteintech).

*L. lactis* culture samples were thawed in an ice bath, briefly sonicated (UPS200S sonicator; Hielscher, Germany) and loaded onto the gel as described above. After the proteins were transferred to a nitrocellulose membrane, the membrane was blocked in 5% skim dried milk in TBST and incubated overnight at 4 °C in 5% skim dried milk in TBST with the following two rabbit polyclonal antibodies: FLAG-tag (1:1000; Proteintech, Rosemont, IL, USA) or anti-CNL (1:2000) [29]. Following three washes with TBST, membranes were incubated for 2 h with goat anti-rabbit IgG, Dylight 650 conjugate (1:5000; Thermo Fisher Scientific), or horseradish peroxidase-conjugated goat anti-rabbit IgG (1:5000, Jackson ImmunoResearch, West Grove, PA, USA). Images were acquired using a ChemiDoc MP imaging system (Bio-Rad).

### 2.3. Binding of Recombinant Stx1B by HeLa, HT-29, and Caco-2 Cells

HeLa, HT-29, and Caco-2 cells were seeded onto sterilized coverslips (8 mm diameter #1.5) in 24-well plates at a concentration of 5 × 10^4^ cells/well. When confluence was reached, the medium was aspirated, and 300 μL of fluorescein isothiocyanate (FITC)-labelled Stx1B (5 μg/mL in fresh medium) [24] was added to the well, followed by incubation at 37 °C for 1 h. For controls, only medium, without FITC-labelled Stx1B, was added. Afterwards, each coverslip was gently washed three times with PBS, fixed in 4% paraformaldehyde (Electron Microscopy Sciences, Hatfield, PA, USA) in PBS for 20 min at RT, washed three times with PBS, mounted with a 4′,6-diamidino-2-phenylindole (DAPI)-containing mounting agent (ProLong Gold Antifade Mountant with DAPI; Thermo Fischer Scientific), and visualized by confocal microscopy.

### 2.4. Immunofluorescence Staining

CNL [30] at concentrations of 0.1, 1, and 10 μg/mL was added to the Caco-2-coated coverslips. Following 1 h of incubation, cells were washed with PBS, fixed and permeabilized in 4% paraformaldehyde in PBS for 20 min, and incubated in 0.1% Triton X-100 in PBS for 5 min. Non-specific staining was blocked with 1% bovine serum albumin in PBS for 1 h. CNL was detected by incubating cells with 20 μg/mL of affinity-purified rabbit anti-CNL primary antibody [30] for 1 h followed by a subsequent incubation with the secondary Alexa Fluor 555-conjugated goat anti-rabbit antibody (1:1000; Life Technologies, Carlsbad, CA, USA) for 1 h in 1% bovine serum albumin in PBS. After each step, the cells were washed three times with PBS. Cells incubated with primary and secondary antibodies in the absence of CNL were used as controls for nonspecific binding. Immunostained cells were mounted onto slides with the DAPI-containing mounting agent and visualized by confocal microscopy.

### 2.5. Confocal Microscopy

The slides were imaged with a confocal microscope (LSM-710, Carl Zeiss, Oberkochen, Germany). Images were collected using a 63× immersion oil objective with settings to detect brightfield, DAPI, Alexa 488, and Alexa 647. Images were prepared using the ImageJ software.

### 2.6. Cell Viability Assay

The effect of recombinant lectins on Caco-2 cell viability was assessed with MTS colorimetric assay as described previously [37]. Caco-2 cells were seeded onto 96-well culture plates (3 × 10^4^ cells/well) and treated with increasing concentrations (0.1, 1, 10, 25, 100, and 250 μg/mL) of Stx1B [24] and CNL [30] in complete medium. For controls, medium devoid of lectins was added. The cells were incubated for 24 h and 48 h at 37 °C. Their viability was assessed using the CellTiter 96 Aqueous One Solution Cell Proliferation Assay (Promega, Madison, WI, USA) according to the manufacturer’s instructions. Absorbance was measured with a microplate reader (Tecan) at a wavelength of 492 nm. The experiment was performed in triplicate, and the results were normalized to the controls (mean values of treated versus untreated cells).

### 2.7. Bacterial Strains and Growth Conditions

The bacterial strains used in this study are listed in Table 1. *Escherichia coli* strain DH5α was grown under aeration at 37 °C in lysogeny broth medium (Sigma Aldrich, St. Louis, MO, USA) supplemented with ampicillin (100 µg/mL; Sigma Aldrich, St. Louis, MO, USA). *L. lactis* NZ9000 was grown without aeration at 30 °C in M-17 medium (Merck, Kenilworth, NJ, USA) supplemented with 0.5% glucose (GM-17) and chloramphenicol (10 µg/mL). Biliverdin HCl (15.5 µg/mL; Sigma Aldrich, St. Louis, MO, USA) was added for the expression of infrared fluorescent protein (IRFP).

### 2.8. Molecular Cloning

Plasmid DNA was isolated with NucleoSpin Plasmid (Macherey and Nagel, Düren, Germany), with an additional lysozyme treatment step for *L. lactis*. Competent *L. lactis* cells resuspended in solution of 0.5 M sucrose (Sigma Aldrich, St. Louis, MO, USA) and 10% glycerol (Sigma Aldrich, St. Louis, MO, USA) were transformed with 200–400 ng of plasmid DNA by electroporation (25 µF, 2 kV, 200 Ω) using the Gene Pulser II apparatus (Bio-Rad, Hercules, CA, USA) according to the manufacturer’s instructions (MoBiTec GmbH, Goettingen, Germany). Plasmids were sequenced by Eurofins Genomics (Ebersberg, Germany).

The lectin genes *stx1B* (GenBank accession number: CP050498.1) and *cnl* (UniProt accession number: B2ZRS9, *L. lactis* codon-optimized, encoding non-dimerizing mutant) were amplified from pET28-Stx1B [24] and gBlock (IDT), respectively, by PCR using the primers specified in Table 1. Amplicons were cloned into the pGEM-T Easy plasmid and then transferred to the plasmid pSDBA3b via the BamHI/EcoRI restriction sites to yield pSD-Stx1B and pSD-CNL. FLAG-tag (24 bp) was inserted via NcoI/BamHI as described previously [42]. The whole cassette encoding the spUsp45-Stx1B-acmA3b fusion protein was transferred to the first multiple cloning site (MCS 1) in the dual promoter plasmid pNZDual via the NcoI/XbaI restriction sites. Finally, IRFP was cloned to the second multiple cloning site (MCS 2) of the plasmid pNZDual via the NdeI/XhoI restriction sites, yielding pNZD-Stx1B-IRFP. The cassette encoding the spUsp45-CNL-acmA3b fusion protein was similarly transferred to the MCS 1 in the dual promoter plasmid with IRFP in the MCS 2, yielding pNZD-CNL-IRFP.

### 2.9. The Expression of Stx1B and CNL Fusion Proteins in L. lactis

Overnight cultures of *L. lactis* harboring the appropriate plasmids were diluted (1:100) in 10 mL of fresh GM-17 medium containing chloramphenicol (10 µg/mL) and biliverdin (15.5 µg/mL), and grown to an optical density of A600 = 0.8–1.0. Fusion protein expression was induced with nisin (25 ng/mL; Fluka Chemie AG, Buchs, Switzerland) [38]. After 3 h of incubation at 30 °C, 1 mL of the cultures was stored at 4 °C for flow cytometry analysis. For SDS-PAGE analysis, the remaining cell culture was centrifuged at 5000× *g* for 10 min, and the cell pellets were resuspended in 400 µL of phosphate-buffered saline (PBS; 137 mM NaCl, 2.7 mM KCl, 12.5 mM Na_2_HPO_4_, 1.98 mM KH_2_PO_4_, pH 7.4) and stored at −20 °C. To evaluate binding to cancer cells, *L. lactis* cultures were centrifuged at 5000× *g* for 10 min, washed twice with PBS, resuspended in PBS to an optical density of A_600_ = 0.8, and stored at 4 °C. Before the adhesion assay, *L. lactis* were resuspended in RPMI 1640 medium with L-glutamine and HEPES (Lonza, Basel, Switzerland).

### 2.10. Fluorescence Measurements of IRFPs

Aliquots of cell cultures (200 µL) with an optical density of A600 = 0.8 were transferred to black, flat-bottom 96-well plates (Greiner, Kremsmünster, Austria). Fluorescence was measured on an Infinite M1000 microplate reader (Tecan, Männedorf, Switzerland), with excitation/emission at 690 nm/713 nm. Two technical replicates of the measurements were performed.

### 2.11. Flow Cytometry

*L. lactis* cultures (20 μL) in the stationary growth phase were added to 500 μL of TBS (50 mM Tris-HCl, 150 mM NaCl, pH 7.5) and centrifuged at 5000× *g* for 5 min at 4 °C. The pellets were resuspended in 500 μL of TBS containing rabbit polyclonal anti-FLAG-tag (1:500; Proteintech) or anti-CNL (1:1000) for the detection of Stx1B and CNL, respectively [43]. After 2 h of incubation at room temperature (RT) with constant shaking at 100 rpm, the cells were washed three times with 200 μL of TBS with 0.1% Tween-20 (0.1% TBST) and resuspended in 500 μL TBS containing an Alexa Fluor 488-labelled anti-rabbit antibody (1:2000; Cell Signaling Technology). After 2 h of incubation at RT with constant shaking at 100 rpm, the cells were washed three times with 200 μL of 0.1% TBST and finally resuspended in 500 μL TBS. The samples were analyzed using a flow cytometer (FACS Calibur; Becton Dickinson, Franklin Lakes, NJ, USA) with excitation/emission at 488/530 nm in the FL1 channel. The geometric mean fluorescence intensity of at least 20,000 *L. lactis* cells in the appropriate gate was measured.

### 2.12. L. lactis Cell Adhesion Assay

HeLa, HT-29, and Caco-2 cells were seeded onto sterilized coverslips (8 mm diameter #1.5) in 24-well plates. The seeding concentrations were determined to reach the desired confluence (5 × 10^4^/well for HeLa, 1 × 10^5^/well for HT-29, and 1.5 × 10^5^/well for Caco-2). After 48 h, the medium was aspirated, and 500 μL of the *L. lactis* culture (at A_600_ = 0.8) in RPMI was added to each well. The cells were incubated with *L. lactis* cultures for 2 h at 37 °C with 5% CO_2_. Following incubation, cells were gently washed twice with PBS to remove unattached *L. lactis*, fixed in 4% paraformaldehyde in PBS for 20 min at RT, washed twice with PBS, mounted with the DAPI-containing mounting agent, and visualized by confocal microscopy.

### 2.13. Statistical Analyses

Statistical analyses were performed using the GraphPad Prism 6 software. The data are presented as mean ± standard deviation. Student’s *t*-tests were used to assess significant differences between the lectin-displaying *L. lactis* and respective controls.

## 3. Results

### 3.1. CNL-Binding Glycoproteins in HT-29 and Caco-2 Whole-Cell Lysates

**Figure 1:**
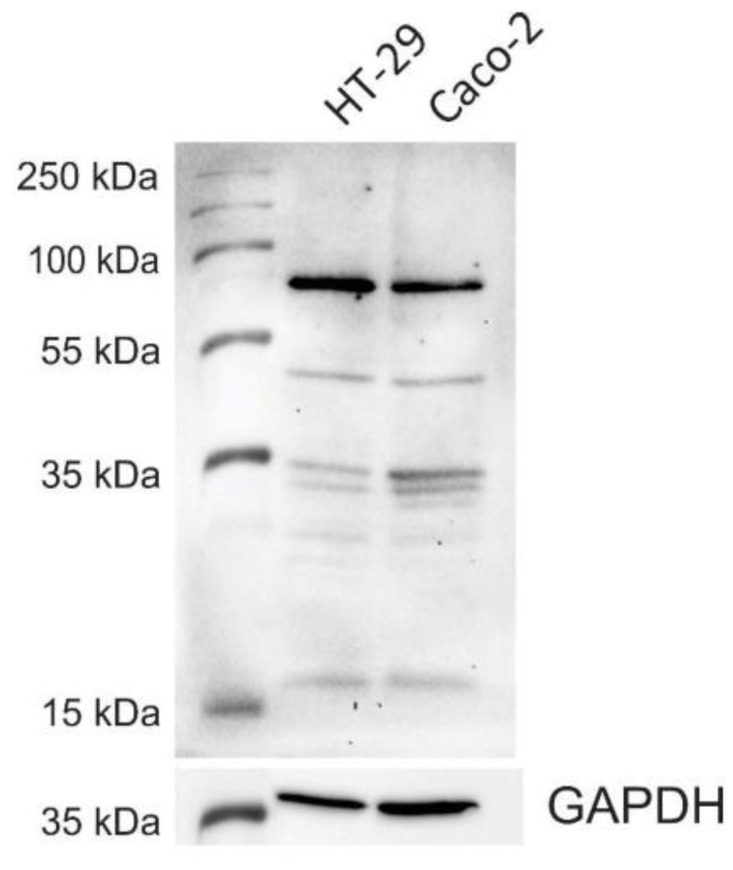
A Western blot showing the *Clitocybe nebularis* lectin-mediated recognition of glycoproteins in HT-29 and Caco-2 cell lysates. GAPDH was used as a loading control.

The presence of the CNL-binding glycoproteins in HT-29 and Caco-2 cells was determined by exposing the blot of whole-cell lysates to CNL and performing detection with anti-CNL antibodies. In both HT-29 and Caco-2 cell lysates, CNL bound to several glycosylated proteins, of which a protein with a molecular weight of 70 kDa exhibited the most CNL binding (Figure 1). A protein of similar size was previously detected in HT-29 cells with the LacdiNAc-specific lectin *Wisteria floribunda* agglutinin [44].

### 3.2. Binding of Recombinant Lectins to HeLa, HT-29, and Caco-2 Cancer Cells

**Figure 2:**
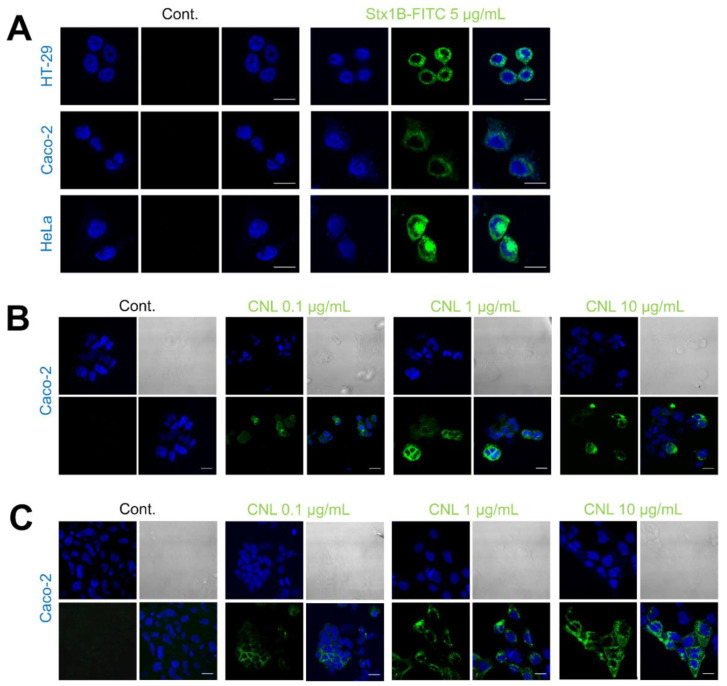
Representative confocal microscopy images showing the binding of FITC-labelled Shiga holotoxin subunit B (Stx1B-FITC; 5 μg/mL) to HT-29, Caco-2, and HeLa cells after 1 h (**A**) and the binding of *Clitocybe nebularis* lectin (CNL; 0.1, 1, or 10 μg/mL), detected by immunostaining, to Caco-2 cells after 1 h (**B**) and 48 h (**C**). (**A**) DAPI (blue; left panel), Stx1B-FITC (green; middle panel), and merged images (right panel). (**B**) DAPI (upper left panel), bright field (upper right panel), CNL detected with rabbit anti-CNL antibody in combination with anti-rabbit Alexa Fluor 555-conjugated antibody (green; lower left panel), and merged images (lower right panel). Scale bars: 20 µm.

The FITC-labelled recombinant protein Stx1B (Stx1B-FITC) was tested for binding to HeLa, HT-29, and Caco-2 cells. Compared to the control, significant binding of Stx1B-FITC was observed, confirming the presence of its corresponding carbohydrate ligand Gb3 in all the cell lines included in the study. The amount of Stx1B-FITC binding to HeLa cells was larger than that to HT-29 and Caco-2 cells (Figure 2A). The binding of recombinant CNL to Caco-2 cells was analyzed with immunocytochemical staining using CNL-specific antibodies. Treatment of Caco-2 cells with CNL at concentrations of 0.1, 1, and 10 μg/mL resulted in dose-dependent CNL binding. After 1 h of incubation, a significant number of Caco-2 cells bound CNL (Figure 2B), and this increased substantially after 48 h of incubation (Figure 2C). No signal was detected in untreated cells labelled with antibody alone.

### 3.3. The Effect of Stx1B and CNL Lectins on Caco-2 Cell Viability

**Figure 3:**
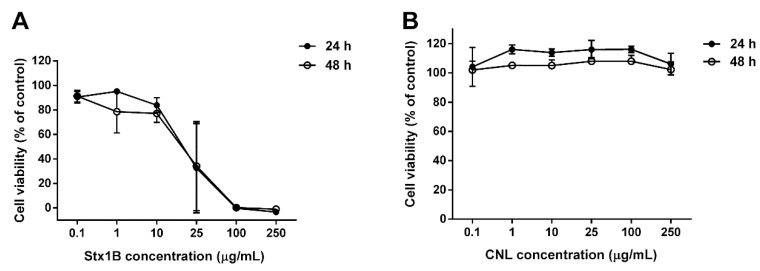
The viability of Caco-2 cells treated with increasing concentrations of Shiga holotoxin subunit B (Stx1B; **A**) and *Clitocybe nebularis* lectin (CNL; **B**) for 24 h (closed circles) and 48 h (open circles). Cell viability is presented as the percentage of viable cells normalized to untreated (control) cells (at 100%). Data are presented as means with error bars indicating standard deviations of triplicate measurements.

The effect of lectins on Caco-2 cell viability was evaluated after 24 and 48 h using the MTS assay. Recombinant protein Stx1B [24] at concentrations of up to 25 μg/mL did not affect cell viability, while higher concentrations decreased the number of viable cells (Figure 3A). Conversely, CNL at concentrations of up to 250 μg/mL exerted no effect on cell viability after 24 and 48 h of exposure (Figure 3B). Of note, the cells were slightly more viable in the presence of CNL; this is in line with experiments analyzing monomeric CNL cytotoxicity in Jurkat human leukemic T cells [30].

### 3.4. Genetic Constructs for Stx1B and CNL Lectin Display on L. lactis

**Figure 4:**
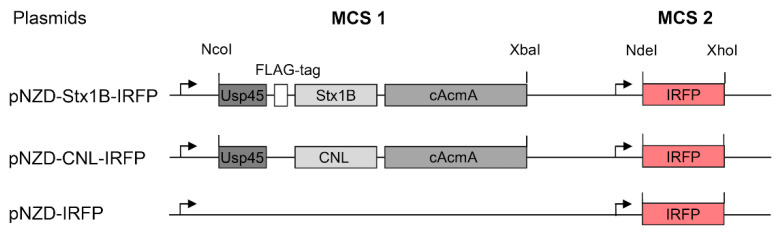
A scheme of the genetic constructs for the display of the Shiga holotoxin subunit B (Stx1B) lectin and *Clitocybe nebularis* lectin (CNL) on the surface of *Lactococcus lactis*.

Two fusion genes were constructed to display lectins on the surface of *L. lactis*. The genes for the lectins Stx1B and CNL were fused with the gene for the Usp45 secretion signal at the 5′-end and with the gene for the non-covalent cAcmA surface anchor at the 3′-end, as described previously [45]. The gene for the FLAG-tag was added for Stx1B detection. The genes were cloned under the control of the NisA promoter into our previously reported dual promoter plasmid pNZDual [41], with two multiple cloning sites (MCS 1 and MCS 2) to enable the simultaneous expression of two fusion proteins. The gene for IRFP was included in MCS 2 to enable the visualization of the *L. lactis*. The fusion genes in pNZDual are listed in Table 1 and shown schematically in Figure 4.

### 3.5. Expression of Lectin Fusion Proteins in L. lactis

**Figure 5:**
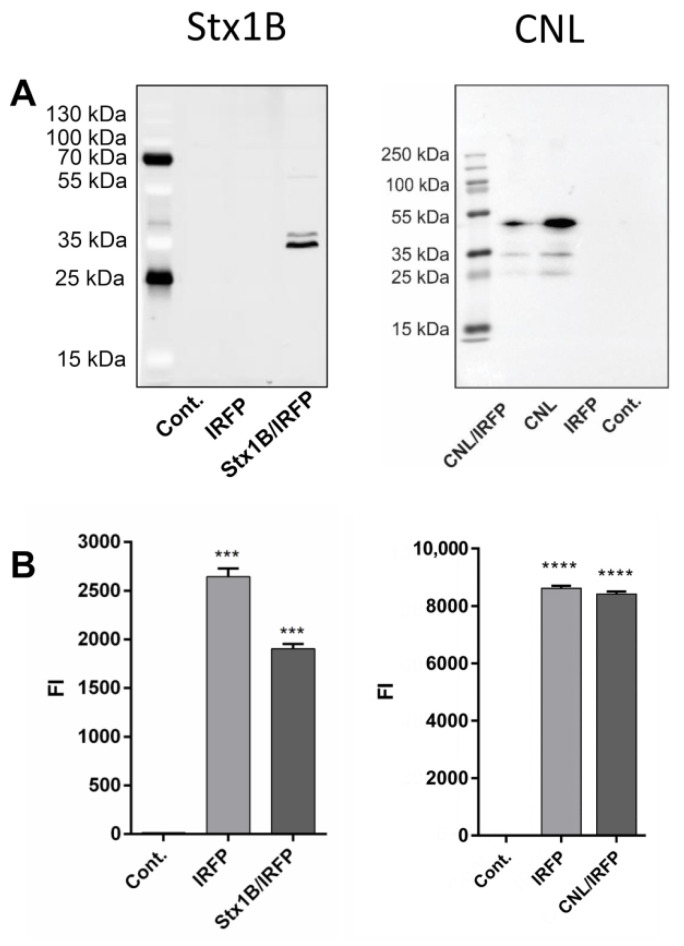
Expression of lectin fusion proteins and infrared fluorescent protein (IRFP) in *L. lactis.* Shiga holotoxin subunit B (Stx1B; left) and *Clitocybe nebularis* lectin (CNL; right) in fusion with Usp45 secretion signal and cAcmA anchoring domain were detected in the cell lysate of *L. lactis* by western blot with the anti-FLAG-tag and anti-CNL antibodies, respectively (**A**). The concomitant expression of infrared fluorescent protein (IRFP) was demonstrated by fluorescence intensity (FI) measurements (**B**). Cont.: control containing empty plasmid pNZ8148. Error bars denote standard deviations. Significant differences were determined by the Student’s *t*-test (*** *p* < 0.001, **** *p* < 0.0001).

Both lectins Stx1B (Uniprot ID: Q7DH26) and CNL (UniProt ID: B2ZRS9) have low molecular weights (Stx1B, 7.7 kDa; CNL, 15 kDa) and are therefore suitable for *L. lactis* expression and surface display. The expression of Stx1B and CNL in fusion with Usp45 secretion signal and cAcmA anchoring domain was confirmed in *L. lactis* cell lysates with SDS-PAGE followed by western blot analysis (Figure 5A) using the anti-FLAG and anti-CNL antibodies, respectively. The size of Stx1B fused to the cAcmA anchoring domain was calculated to be around 34 kDa (FLAG-tag ∼1 kDa, Stx1B ∼7.7 kDa, and cAcmA ∼25 kDa), which corresponds to the size detected in cell lysates of *L. lactis* harboring pNZD-Stx1B-IRFP (Figure 5). The size of CNL fused to the cAcmA anchoring domain was calculated to be around 40 kDa (CNL monomer ∼15.9 kDa and cAcmA ∼25 kDa), which is somewhat smaller than the size (∼45 kDa) detected in cell lysates of *L. lactis* harboring pNZD-CNL-IRFP (Figure 5). No bands were detected in control *L. lactis* harboring IRFP-containing plasmid or empty plasmid (pNZ8148). The concomitant expression of IRFP was confirmed by fluorescence intensity measurements in both lectin-displaying *L. lactis* strains (Figure 5B). The level of IRFP co-expressed in the Stx1B-displaying strain was somewhat lower than that expressed in the control strain. Conversely, the CNL-displaying strain expressed a similar amount of IRFP as the control strain.

### 3.6. Surface Display of Lectins on L. lactis

**Figure 6:**
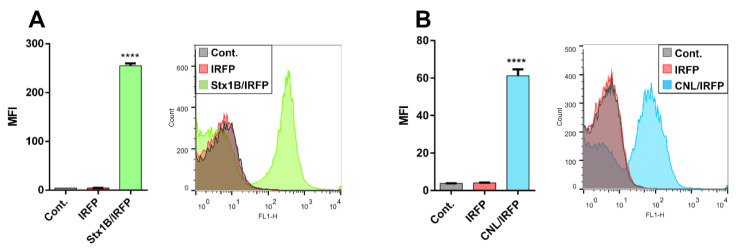
Flow cytometry of *L. lactis* displaying Shiga holotoxin subunit B (Stx1B; **A**) and *Clitocybe nebularis* lectin (CNL; **B**), detected with the anti-FLAG and anti-CNL antibodies, respectively. Mean fluorescence intensity (MFI) and representative histograms of three measurements are depicted. Cont.: *L. lactis* containing an empty plasmid pNZ8148; IRFP: *L. lactis* containing pNZD-IRFP. Significant differences were determined by the Student’s *t*-test (**** *p* < 0.0001).

The statistically significant surface display of lectins on *L. lactis* was confirmed with flow cytometry. A characteristic shift in mean fluorescence intensity was observed for Stx1B-displaying and CNL-displaying strain in comparison to control *L. lactis* harboring empty plasmid or pNZD-IRFP (Figure 6). Flow cytometry also verified that lectins were accessible on the *L. lactis* cell surface.

### 3.7. The Adhesion of Lectin-Displaying L. lactis to Cancer Cells

**Figure 7:**
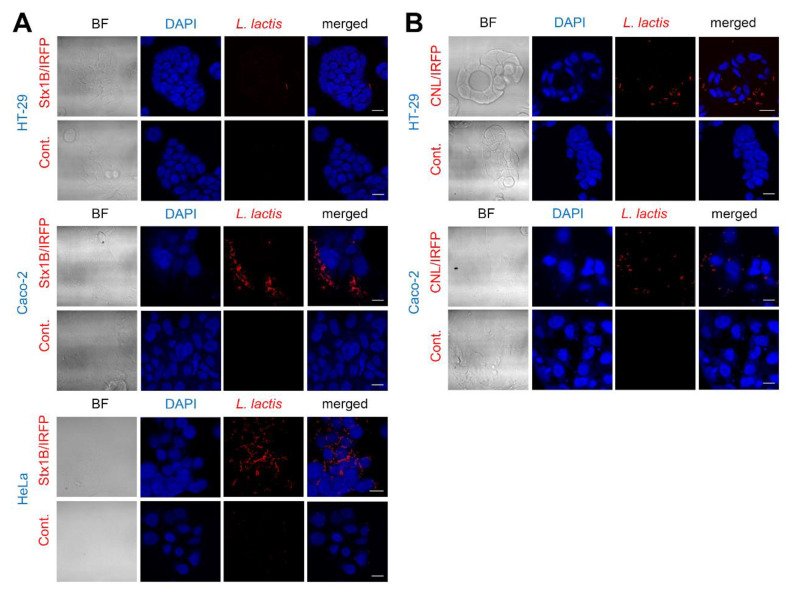
Representative confocal microscopy images showing binding of Shiga holotoxin subunit B (Stx1B)-displaying *L. lactis* to HeLa, HT-29, and Caco-2 cells (**A**) and *Clitocybe nebularis* lectin (CNL)-displaying *L. lactis* to HT-29 and Caco-2 cells (**B**). Cont.: control *L. lactis* expressing infrared fluorescent protein (IRFP); BF: bright field. Scale bars: 20 µm.

The binding of Stx1B-displaying *L. lactis* to cancer cells was analyzed. A large number of Stx1B-displaying *L. lactis* adhered to HeLa cells. A significant number of Stx1B-displaying *L. lactis* clusters also adhered to Caco-2 cells, whereas only a few *L. lactis* clusters interacted with HT-29 cells (Figure 7A). The ability of CNL-displaying *L. lactis* to adhere to HT-29 and Caco-2 cells was also examined. A modest number of CNL-displaying *L. lactis* directly interacted with HT-29 and Caco-2 cells grown in the form of islets (Figure 7B). Most of the *L. lactis* adhered to the outer edges of the larger islets, while few *L. lactis* adhered to the inner areas of the islets. Some individual cancer cells and small islets appeared surrounded by the adhered CNL-displaying *L. lactis*. No *L. lactis* were visible in control cell cultures incubated with IRFP-expressing *L. lactis*, which demonstrates that *L. lactis* without lectin displayed on their surface do not bind to cells.

## 4. Discussion

In this study, we utilized lectins to target *L. lactis* to cancer cells via their interaction with glycans on the cancer cells’ surface. Various cancer tissues produce oligosaccharides that differ from the glycosylation patterns in non-malignant tissue. Lectins possess a high level of specificity for the tumor-associated carbohydrates and are therefore considered for selective delivery of anticancer agents to tumors. For this purpose, we constructed two recombinant *L. lactis* strains that displayed the Gb3-recognizing lectin Stx1B and LacDiNAc-recognizing lectin CNL on their surface. We demonstrated adhesion of these lectin-displaying *L. lactis* onto HT-29, Caco-2, and HeLa cancer cells.

First, the colorectal cancer cell lines HT-29 and Caco-2 were tested for the expression of the Gb3 receptor (for Stx1B), while HeLa cells were included as a control, as they are known to express Gb3 and bind Stx1B. We confirmed successful Stx1B-FITC binding to all three cell lines tested. HeLa cells exhibited the largest amount of binding, which is in agreement with the previously observed high expression of the Gb3 receptor in this cell line [46,47]. Less binding was observed to the tested colorectal cell lines, which is also in accordance with previously reported data [48,49].

Unlike for the Gb3 receptor, data on the expression of the CNL target LacdiNAc in HT-29 and Caco-2 cells is scarce. In HT-29 cells, LacdiNAc has been demonstrated to be the ligand for macrophage galactose-type lectin [50] and *Wisteria floribunda* agglutinin lectin [51], whereas the presence and identity of CNL-binding glycoproteins remain largely unknown. We, therefore, performed western blotting of HT-29 and Caco-2 whole-cell lysates, probed them with CNL, and analyzed the interaction of CNL with glycoproteins from the lysates. Multiple bands were detected, suggesting that CNL recognizes more than one glycoprotein in the lysate of tested cells. Although their identity is not yet known, we found that a 70 kDa glycoprotein, which exhibited the highest CNL binding, was also previously detected in HT-29 cells with the LacdiNAc-specific lectin *Wisteria floribunda* agglutinin [45]. We further examined whether CNL binds to intact Caco-2 cells via its target glycoproteins. Immunocytochemical analysis with specific anti-CNL antibodies showed successful dose- and time-dependent accumulation of CNL on the surface of Caco-2 cells. The substantial binding was achieved at 1 µg/mL of CNL. However, the selectivity of CNL for Caco-2 and HT-29 cells could not be assessed due to the lack of appropriate control (cell line with confirmed absence of LacdiNAc).

To test the effect of the recombinant lectins Stx1B and CNL on Caco-2 cell viability, we performed a colorimetric MTS assay. Cell viability was assessed after 24 and 48 h incubations with increasing concentrations of the recombinant lectins. CNL has been previously shown to have an antiproliferative effect on human leukemic T lymphocytes (Jurkat cells), decreasing cell viability by >50% at the highest concentration (100 µg/mL) [30]. The effect was dependent on bivalent binding of homodimeric CNL to cell-surface carbohydrates. In this study, we used a non-dimerizing CNL, which caused no effect on Caco-2 cell viability, even at the relatively high concentration of 250 μg/mL. Although limited to 48 h incubation, our results substantiate previous findings regarding a lack of cytotoxicity of non-dimerizing CNL mutant against Jurkat cells [30] and reinforce the conclusion that monomeric CNL is generally non-toxic. Conversely, recombinant Stx1B decreased cell viability at concentrations above 25 µg/mL, consistent with a previous observation that Stx1B is capable of inducing apoptosis even in the absence of the toxic subunit StxA [11]. This is also in agreement with the measured apoptotic effect in HeLa and Vero cells in which recombinant Stx1B concentrations of >20 µg/mL significantly decreased cell viability [10].

After verifying the presence of lectins’ target sites on HeLa, HT-29 and Caco-2 cells, we displayed Stx1B and CNL, individually, on the surface of *L. lactis* and assessed the adhesion of engineered bacteria on target cancer cells. To achieve surface display on *L. lactis*, Stx1B and CNL were fused with an Usp45 secretion signal and cAcmA anchoring domain. IRFP was co-expressed for the purpose of detection and visualization. A significant level of Stx1B and CNL expression and surface display on *L. lactis* was verified by western blotting and flow cytometry, respectively. Multiple bands, observed by western blotting, were probably the consequence of cAcmA degradation and could be prevented by using *L. lactis* NZ9000ΔhtrA strain as previously reported [22]. The co-expression of IRFP was confirmed by fluorescence measurements.

Finally, we evaluated the binding of engineered Stx1B and CNL lectin-displaying *L. lactis* to cancer cells. For Stx1B-displaying *L. lactis*, adhesion assays were performed on HeLa, HT-29, and Caco-2 cells. While only a few Stx1B-displaying *L. lactis* cells adhered to HT-29 cells, many *L. lactis* clusters attached to the surface of Caco-2 and HeLa cells. The extent of *L. lactis* binding was greater in Caco-2 cells than that in HT-29 cells, which is surprising as HT-29 cells were reported to express more Gb3 than Caco-2 cells [49]. Nevertheless, the binding of Stx1B to Caco-2 cells has already been demonstrated [27], which substantiates our observations. The binding of CNL-displaying *L. lactis* to HT-29 and Caco-2 cells was also assessed. CNL-displaying *L. lactis* adhered to HT-29 and Caco-2 cells. As a control, *L. lactis* that did not display lectins was used and did not adhere to cancer cells, confirming that the binding to the cancer cell surface is lectin-mediated. More Stx1B-displaying *L. lactis* adhered to Caco-2 cells than to HT-29 cells; conversely, more CNL-displaying *L. lactis* adhered to HT-29 cells than to Caco-2 cells. These observed differences in cell specificity might be attributed to the different expression levels of target receptors on the surface of cancer cells. Apart from that, efficient ligand–receptor interaction for active targeting depends on variety of factors, including the availability of the receptor on the cell surface, the rate of internalization over shedding of the surface receptor following ligand binding and the affinity of the ligand for the receptor [52]. Overall, both Stx1B- and CNL-displaying *L. lactis* showed a weak to moderate level of adhesion to HT-29 and Caco-2 cancer cells in comparison to the high level of adhesion of Stx1B-displaying *L. lactis* to HeLa cells. Nevertheless, this study has demonstrated that lectin-mediated targeting of bacteria to the cancer cells is feasible. Moreover, a further increase in cell adhesion could be accomplished by testing additional lectins or by displaying multiple lectins, including those with cytoadhesive characteristics, such as wheat germ agglutinin that was shown to improve association of drug-loaded polymeric nanospheres to Caco-2 cells [53]. The engineered, lectin-functionalized, bacteria would be particularly suitable for delivery of therapeutic proteins to the gastrointestinal tract via oral route for the treatment of colorectal cancer, thanks to the ability of *L. lactis* to survive harsh conditions in gastrointestinal tract, as well as the stability of lectins in gastro-intestinal fluids and their resistance to degradation by digestive processes [54].

## 5. Conclusions

This is the first report on lectin-displaying bacteria engineered for glycan-targeting cancer therapy. Two lectins that target tumor glycoproteins were displayed on the surface of *L. lactis*. The ability of lectin-displaying *L. lactis* to specifically adhere to the surface of cancer cells and the ability of recombinant lectins to bind to these cells was demonstrated. Taken together, our results suggest that lectins, displayed on *L. lactis*, can achieve active targeting of cancer cells via their interactions with glycans on the surface of cancer cells. The use of lectins as potential drug carriers has been previously shown, and this study demonstrates that their applicability could be broadened by their combined use with live food-grade bacteria.

## Figures and Tables

**Table 1:** Strains, primers, and plasmids used in this study.

Strain, Primer, or Plasmid	Relevant Features or Sequence	Reference
Strains		
*E. coli* DH5α	endA1 glnV44 thi-1 recA1 relA1 gyrA96 deoR F^−^ Φ80d*lacZ*ΔM15 Δ(*lacZYA-argF*)U169, hsdR17(r_K_^−^ m_K_^+^), λ–	Invitrogen
*L. lactis* NZ9000	MG1363 *nisRK* Δ*pepN*	NIZO
Primers		
Stx1B-F-Bam	5′-AGGATCCAAAAAAACATTATTAATAGCTGCATC-3′	This work
Stx1B-R-Eco	5′-AGAATTCACGAAAAATAACTTCGCTG-3′	This work
CNL-F-Bam	5′-TGGATCCTCTATTACACCTGGTACTTATAATATTAC-3′	This work
CNL-R-Eco	5′-AGAATTCTACGGCAGAGACACTTTC-3′	This work
Genes		
CNL	GGATCCTCTATTACACCTGGTACTTATAATATTACAAATGTTGCTTAT ACAAACAGATTGATTGATTTGACAGGTAGTAATCCTGCTGAAAATA CACTTATTATCGGTCATCATCTTAACAAAACACCTTCAGGTTATGGA AATCAACAGTGGACACTTGTCCAGCGACCACACACAACTATCTATA CTATGCAAGCAGTTAATCCACAATCTTATGTACGAGTTCGTGATGAT AATTTAGTTGACGGAGCAGCACTTGTAGGAAGTCAACAGCCTACAC CTGTCAGTATTGAATCAGCCGGAAATTCAGGTCAATTTCGAATTAAA ATTCCAGATTTAGGTTTAGCTTTAACTTTACCTTCAGACGCAAATAGT ACTCCTATTGTACTTGGAGAAGTTGATGAAACATCTACTAATCAATTG TGGGCATTTGAAAGTGTCTCTGCCGTAGAATTC	This work
Plasmids		
pGEM-T Easy	Ap^r^, cloning vector for PCR products	Promega
pET28-Stx1B	pET28b containing Stx1B gene	[24]
pNZ8148	pSH71 derivative, P*_nisA_*, Cm^r^, nisin-controlled expression	[38,39,40]
pSDBA3b	pNZ8148 containing gene fusion of *sp*_Usp45_, *b-dom*, and *acmA3b*	[22]
pSD-Stx1B	pNZ8148 containing gene fusion of *sp*_Usp45_, *flag, stx1B*, and *acmA3b*	This work
pSD-CNL	pNZ8148 containing gene fusion of *sp*_Usp45_, *cnl*, and *acmA3b*	This work
pNZDual	pNZ8148 containing two multiple cloning sites (MCS1 and MCS2)	[41]
pNZD-IRFP	pNZDual with *irfp* in MCS2	[41]
pNZD-Stx1B	pNZDual with *flag-stx1B* in MCS1	This work
pNZD-CNL	pNZDual with *cnl* in MCS1	This work
pNZD-Stx1B-IRFP	pNZDual with *flag-stx1B* in MCS1 and *irfp* in MCS2	This work
pNZD-CNL-IRFP	pNZDual with *cnl* in MCS1 and *irfp* in MCS2	This work
